# The role of autophagy in the pathogenesis and treatment of amyotrophic lateral sclerosis (ALS) and frontotemporal dementia (FTD)

**DOI:** 10.1080/27694127.2025.2474796

**Published:** 2025-03-20

**Authors:** Jimmy Beckers, Philip Van Damme

**Affiliations:** a Department of Neurosciences, Experimental Neurology, and Leuven Brain Institute (LBI), KU Leuven-University of Leuven, Leuven, Belgium; b Department of Neurology, University Hospitals Leuven, Leuven, Belgium

**Keywords:** Amyotrophic lateral sclerosis (ALS), autophagy, frontotemporal dementia (FTD), lysosome, autophagosome, endosome, endolysosome, neurodegeneration

## Abstract

Amyotrophic lateral sclerosis (ALS) and frontotemporal dementia (FTD) represent two extremes of a neurodegenerative disease spectrum characterised by overlapping genetic, clinical, and neuropathological features. This review covers the intricate relationship between both ALS and FTD and defects in the autophagy and endolysosomal pathway as recent evidence has pointed towards alterations in these pathways as being a root cause of disease pathogenesis. Here, we review the current knowledge on the interplay between ALS/FTD and lysosomebased proteostasis pathways and carefully asses the steps of the autophagy and endolysosomal pathways that are impaired by ALS or FTDcausing variants. Finally, we present a comprehensive overview of therapeutic strategies aimed at restoring autophagic and lysosomal function as potential avenues for mitigating the impact of these devastating diseases. Through this review, we aim to enhance the understanding of the pathophysiological mechanisms involving autophagy and/or the endolysosomal system that underlie the ALS-FTD spectrum and underscore the necessity for specific therapeutic approaches that target these shared vulnerabilities.

## Introduction

### The ALS-FTD disease spectrum

Amyotrophic lateral sclerosis (ALS) and frontotemporal dementia (FTD) are both fatal and progressive adult-onset neurodegenerative diseases [1,2]. The genetic, clinical and neuropathological overlap of ALS and FTD is in line with anALS/FTD disease spectrum with pure ALS and FTD at the extremes, and intermediate phenotypes distributed throughout the spectrum [3-5]. Multiple gene variants are reported to be causal for both ALS and FTD. Examples of these genes include *VCP* (valosin containing protein), *TBK1* (TANK binding kinase 1), *FUS* (FUS DNA binding protein), *TARDBP/TDP-43 (*TAR DNA binding protein) and most commonly *C9orf72* (chromosome 9 open reading frame 72) [3,4]. While in ALS, loss of both upper and lower motor neurons (MNs) located in the motor cortex, brainstem and spinal cord results in progressive paralysis and muscle wasting, FTD, is characterised by degeneration of frontal and/or anterior temporal lobes of the brain resulting in multiple deficits in language, personality or behaviour [1,2,4,6-8]. In approximately 35-40% of FTD patients and in about 5-10% of ALS patients, a familial history of the disease, usually inherited with an autosomal dominant Mendelian pattern, is present. These familial forms of the disease (fALS and fFTD) cannot be clinically distinguished from their sporadic variants (sALS and sFTD respectively) [2,4,5,7-12]. Disease onset in ALS and FTD typically starts in late midlife although both neurodegenerative disorders are characterised by a high degree of heterogeneity and variability in the disease manifestation, heritability and disease duration, the latter ranging on average from two to five years after symptom onset in ALS and three to eleven years after onset in FTD [7,8,13,14].

Both disorders are also linked at the neuropathological level by the presence of glial and neuronal proteinaceous inclusions with similar composition. In the large majority of ALS patients (~97%) and in roughly 50% of patients with FTD these aggregates stain positive for TDP-43 while aggregates containing FUS are also detected in a subset of both ALS and FTD patients [3,4,15]. About 50% of FTD patients have tau pathology, this subtype is distinct from the TDP-43 related ALS-FTD spectrum and not covered in this review. Apart from TDP-43 pathology, patients with C9orf72 ALS/FTD also present with additional aggregates containing the toxic dipeptide repeat proteins (DPRs) produced from the hexanucleotide repeat expansion (HRE) in the *C9orf72* gene. These TDP-43 inclusions, as well as DPR aggregates, commonly stain positive for SQSTM1/p62, which is an autophagy receptor protein [7,16]. Moreover, while these inclusions immunopositive for TDP-43 and p62 also stained positive for ubiquitin in most ALS cases [17], ubiquitin-negative inclusions have also been reported, especially in white matter lesions of FTD and ALS/FTD patients [18-20]. While at the neuropathological level, both ALS and FTD are well characterised, the disease mechanisms underlying ALS/FTD remain incompletely understood, even despite decades of dedicated research. Moreover, treatment options for patients with ALS and FTD are limited and although more than 60 different drugs covering multiple affected pathways have been tested in clinical trials, only two of them have been approved for clinical use by the FDA and EMA: riluzole for ALS [21-24] and QALSODY® (Tofersen) for the treatment of patients with ALS due to mutations in the *SOD1* gene [21,24,25]. Although Tofersen has a significant impact on the disease progression, its use is limited to patients with a *SOD1* mutations. As such, multidisciplinary care focused on symptom management,quality of life and respiratory and nutritional support, remains the cornerstone in ALS/FTD clinical care [8,21,26,27].

### The intricate relationship between autophagy, the endolysosomal pathway and neurodegeneration

Protein misfolding, resulting in pathological protein inclusions in glia and neurons, such as α-synuclein in PD, amyloid-β plaques and tau tangles in AD, huntingtin in HD, Tau, TDP-43 and FUS in FTD, and TDP-43, SOD1, and FUS in ALS, is considered a hallmark of neurodegenerative diseases (NDs), including ALS and FTD [28-31]. Hence, NDs are also commonly referred to as proteinopathies. These aggregates likely arise from increased wild-type or mutant protein levels due to disrupted autoregulation, protein misfolding, or impaired degradation via cellular proteostasis pathways [30,31]. Proteostasis, a contraction of the words protein and homoeostasis, is essential for maintaining neuronal health and denotes the finely tuned balance of protein concentrations within cells, mainly regulated through the proteasome, autophagy, and endolysosomal pathways [30,31]. Recent genetic, biochemical, and pathological insights across a broad spectrum of these NDs suggest that disturbances in cellular proteostasis may be the primary underlying factor driving disease onset and progression [28-33]. Moreover, the role of proteostasis dysfunction in these diseases extends beyond merely the turnover of aggregation-prone proteins or proteinaceous inclusions, profoundly impacting cellular processes such as inflammation, protein translation, energy metabolism, and various signalling cascades [34-36]. Due to the widespread accumulation of protein aggregates in ALS/FTD and related NDs, multiple groups have studied the potential of compounds that promote the clearance of these neurotoxic protein by enhancing proteostasis in the treatment of these diseases with varying degrees of success [37,38]. Among the various factors that increase the risk of NDs, ageing has by far the most significant impact, as reviewed by [39] and [40]. This is particularly important because, with ageing, the capacity of the cell to sustain proteostasis gradually collapses. Especially in neurons, the progressive decline of both autophagosome biogenesis and maturation, and the concomitant reduction of basal autophagic capacity with ageing, inherently leads to the build-up of immature and morphologically abnormal autophagic vesicles [41-44]. As a result, even healthy ageing is correlated with the widespread aggregation of non-disease proteins [40,45]. Diving deeper into this idea, and despite the general consensus on the toxicity of protein aggregation, the formation of larger protein aggregates might help mitigate cellular damage caused by toxic species of the mutated protein and/or promote their clearance through processes such as aggrephagy [46-48]. In addition, liquid-liquid phase separation of RNA-bindings proteins (RBPs) such as TDP-43, but also FUS, is actually an essential physiological process implicated in the formation of biomolecular condensates with various functional roles in cells [49]. This process can increase the local concentration of certain proteins which may either increase or inhibit their activity. Moreover, phase separation may also impact mesoscale organisation of cellular activity as seen in nucleoli involved in DNA damage repair, stress granules involved in the cellular stress response and transport granules that facilitate the transport and consequent local translation of mRNA (reviewed in [49]). However, aberrant phase transition of TDP-43, FUS, but also other ALS/FTD-related RBPs such as hnRNPA1/2, and TIA1 leads to protein aggregation and disrupts their regular cell function [49,50]. Taken together, phase separation is crucial for several cellular processes, yet when this phase separation process goes awry, it could lead to protein aggregation, a pathological hallmark in both ALS and FTD as well as other NDs [50,51].

Although an age-related decline in proteostasis seems to be present in every cell type, neurons appear to be especially susceptible to this kind of cellular stress. This is not surprising since neurons are non-dividing postmitotic cells that are particularly vulnerable to DNA damage and proteotoxic stress, as they cannot use cell division coupled with programmed cell death to effectively dilute or remove toxic protein aggregates [28,52].

Additionally, neurons are incredibly large cells with a highly polarised morphology due to their extremely long axons. Besides defects in the nucleus and cell body of neurons, axonopathies (i.e. detrimental changes in the axon) have routinely been described in ALS and FTD. More so, axonal degeneration has been proposed to precede the degeneration of neuronal cell bodies, a paradigm put forward as the “dying-back hypothesis” [53,54]. This hypothesis suggests that neuronal pathology starts at the distal ends of the neurons, a process characterised by synaptic dysfunction after which it propagates (“dies-back”) to the MN cell body. During this process, the neurons gradually lose their connection to the muscle (i.e. neuromuscular junction (NMJ)), retract their axons and degenerate [53,54]. This unique neuronal morphology does not only result in a high metabolic demand, but also implies a distinct and characteristic cell-type-specific regulation of the neuronal autophagy-lysosome system [55-60]. For a detailed description of the particularities of axon-specific regulation of the autophagic and endolysosomal pathways we refer the reader to the following reviews [55-60]. The final, and perhaps most convincing, piece of evidence directly linking autophagy dysfunction to neurodegeneration comes from genetics. Deletion of either Atg5, Atg7 or FIP200, three essential autophagic proteins, in mouse neurons results in a neurodegenerative phenotype accompanied by inclusion bodies containing polyubiquitinated proteins and mutations in several proteins involved in autophagy have been associated with ALS-FTD [61-63].

### Evidence for a role of autophagy disruption in the pathogenesis of ALS and FTD

**Figure 1.**
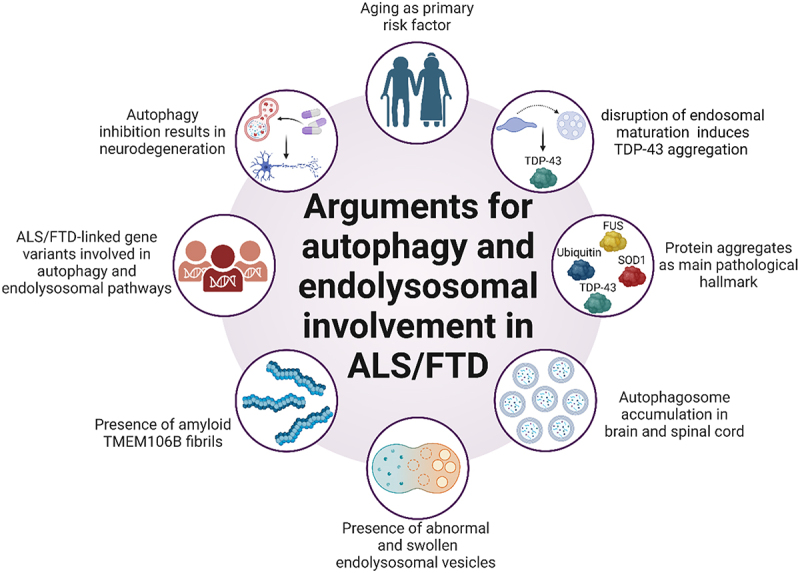
Schematic overview of the various signs that link ALS and FTD to defects in the autophagy and endolysosomal pathways. Abbreviations: ALS: amyotrophic lateral sclerosis; FTD: frontotemporal dementia; TDP-43: TAR DNA binding protein; TMEM106B: transmembrane protein 106B.

Although we will provide a brief and comprehensive explanation of autophagy and the endolysosomal system in the upcoming section, extensive overviews of these pathways lie beyond the scope of this review and are already described in detail elsewhere [43,64-69]. Autophagy, which in this review is referring to macroautophagy, is an essential and highly evolutionary conserved cellular homoeostasis pathway that is responsible for both the bulk and non-selective or selective degradation of organelles and proteins [43,64]. In the simplest sense, autophagy is a lysosome-dependent multi-step process where autophagosomes are formed that engulf and sequester cytoplasmic cargo targeted for degradation (Figure 1) [69,70]. Acidification of these autophagosomes matures them into autolysosomes where their content gets degraded and recycled. Activation of autophagy initiation canonically happens via either activation of the AMPK (AMP-activated protein kinase) or inhibition of the mTORC1 (mechanistic target of rapamycin kinase complex 1) which results in dephosphorylation and the concomitant activation of the ULK1 (unc-51 like autophagy activating kinase 1) protein complex [69,71,72]. Subsequently, the ULK1 complex phosphorylates and activates Beclin1 [73] and Atg14L [74], two key proteins in the PtdIns3K (class III phosphatidylinositol 3-kinase) complex which, in turn, promotes the nucleation of a pre-autophagosomal structure also known as omegasome. This structure then further matures into a phagophore by insertion of LC3-II, the mature and lapidated isoform of MAP1LC3/LC3 (microtubule associated protein 1 light chain 3) and fusion of membranous domains derived from either the plasma membrane, ER, endosomes or Golgi network a process guided by several ATG (autophagy-related) proteins [64,70,75-77]. Finally, autophagosome formation ends when the phagophore closes upon itself thereby sequestering and essentially trapping a part of the cytoplasm and the proteins/organelles it contains [43,70,76,78]. While it was previously thought that this cargo sequestration process was a passive and non-selective process, it is now recognised that autophagy can also be selective by using selective autophagy receptors (i.e. SQSTM1/p62 (sequestome1), OPTN (optineurin), NBR1 (neighbour of BRCA1) that can recognise and deliver specific cargoes (i.e. aggregates, mitochondria, lysosomes) to the phagophore [79-82]. Transport of these autophagosomes along the microtubular network then orchestrates fusion with lysosomes or (late) endosomes thereby respectively forming autophagolysomes or intermediate amphisomes that further mature into autolysosomes by additional fusion events with lysosomes [69,70]. These fusion events are mediated by multiple endolysosomal proteins such as the HOPS (homotypic fusion and protein sorting) complex, LAMP1, Rab7 and a plethora of SNARE (soluble N-ethylmaleimide-sensitive-factor attachment protein receptor) proteins. For further details on the cellular autophagosome-lysosome fusion machinery [83,84] and the transcriptional regulation of autophagy and lysosomal homoeostasis [85-87] we refer to following detailed reviews. Finally, upon maturation, the original autophagosomal membrane along with its content gets degraded by lysosomal hydrolase enzymes which are then actively exported to the cytoplasm and recycled [43,78,88].

While autophagy and the endolysosomal pathway are intricately intertwined by the fusion of endosomes/lysosomes with autophagosomes, the latter also stands on its own [67,76,89,90]. In brief, the endolysosomal system is an intracellular trafficking pathway that internalises material present at the plasma membrane via early endosomes. Subsequently, these vesicles either turn into recycling endosomes that transport cargo back to the plasma membrane or into endosomes that further mature into late endosomes and eventually endolysosomes (Figure 1). Upon degradation of its content, the endolysosomes condense down and reform lysosomes [67,89,91]. This maturation process is orchestrated by multiple Rab GTPases that act in a cascade fashion whereby each previous Rab catalyzes conversion to the next GTPase (reviewed by [92]). As a result, each endosomal subpopulation has its characteristic Rab GTPase population i.e. early endosomes contain Rab5, late endosomes contain Rab7, and recycling endosomes contain either Rab11 or Rab4. In order for endosomes to mature into endolysosomes, they must internalise vesicles derived from the Golgi network containing hydrolytic enzymes. Targeting of these vesicles to the endosomal network uses M6PRs (mannose-6-phosphate receptors) and after an internalisation process mediated by the ESCRT (endosomal sorting complex required for transport) complex, M6PR-containing vesicles are recycled back to the Golgi network by Rab9 and the retromer complex [91-93].

As mentioned previously, TDP-43 inclusions are a key pathological feature in ALS and FTD pathogenesis. While the exact mechanisms that drive TDP-43 aggregation are still under investigation, more and more evidence is pointing towards disruptions of endolysosomal and autophagy pathways and the concomitant reduced clearance of misfolded and/or cytoplasmic TDP-43 species as causal or at least contributing factors in this process [67,90,94-99]. First, both lysosome-dependent pathways are able and needed to regulate basal levels of TDP43 [94,100-102]. In fact, a recent study even showed TDP-43 accumulations specifically in massively enlarged multivesicular bodies (MVBs) [103]. Second, despite not being able to induce TDP-43 aggregation from scratch, inhibition of autophagy has been shown to aggravate TDP-43 aggregation in cells already containing small inclusions [104,105]. Interestingly, genetic or pharmacological disruption of endosomal maturation on the other hand was sufficient to induce TDP-43 aggregation in mice, underscoring the importance of not only autophagy, but also endolysosomal pathway disruptions to drive pathogenesis in TDP-43 proteinopathies [106]. Third, in the same mouse model that harboured mutations in two ALS/FTD genes *C9orf72* and *TBK1*, both gene mutants acted in concert to impair endosome maturation and drive neuronal TDP-43 pathology which coincided with the presence of enlarged endosomes [106,107]. Fourth, targeting the lysosome directly by disrupting its fusion and acidification in iPSC-derived MNs also proved to induce TDP-43 inclusions [108].

In addition to the indirect evidence involving TDP-43 aggregation as stated above, careful analysis of *post-mortem* samples from ALS/FTD patients revealed dysregulation across different stages of the endolysosomal and autophagy pathways. A clear indicator of autophagic defects underlying disease pathogenesis is the autophagosome accumulation found in spinal cord MNs from ALS patients [109]. Evidence for defects in the endosomal recycling pathway are also present as both the endosomal recycling factor Rab11 [110] and components of the retromer complex [111] were found to be reduced in spinal cord tissue of sALS patients. Moreover, abnormal and swollen endolysosomal species are detected in tissue from patients harbouring either ALS, FTD or ALS/FTD-causing mutations [106,112-114]. Intracellular amyloid fibrils containing the lysosomal protein TMEM106B (transmembrane protein 106B), an important regulator of lysosome function, have recently been found in various NDs including ALS and FTD [115-117]. Finally, the identification of several pathogenic gene variants underlying ALS and FTD in the last decades are found to be implicated in multiple aspects of the autophagy and endolysosomal pathways (summarised in Table 1) [67,68,118-120]. Mutations in some of these genes such as on the one hand *SQSTM/p62* (sequestome 1), *OPTN* (optineurin), *UBQLN2* (ubiquilin 2), *TBK1* and on the other hand *GRN* (granulin), *VCP, CHMP2B* (charged multivesicular body protein 2b), *TMEM106B* have a clear link to the lysosomal network causing endolysosomal and selective autophagy defects respectively. Others such as *C9orf72, TARDBP, KIF5A* (kinesin heavy chain isoform 5A), *DCTN1* (dynactin 1), *TUBA4A* (Tubulin α4A chain) and *FUS* seem to have a more complex and indirect relationship with lysosomal pathways impacting processes such as axonal transport and RNA homoeostasis. In the following sections of this review, we will provide an update on the current evidence that links ALS/FTD-associated genes to disruptions in the endolysosomal and autophagy systems and discuss the ongoing therapeutic strategies that target these pathways in ALS and FTD (Table 1).

**Table 1.** Overview of genes classified as causative or risk factors for ALS and/or FTD.

Gene	Encoded protein	Locus	Disease phenotype	Function in autophagy and influence of pathogenic variant	Refs (still import via endnote)
*TBK1*	TANK-binding kinase 1	12q14.2	ALS	Autophagy	[130,131]
*C9orf72*	Chromosome 9 open reading frame 72	9p21.2	ALS, ALS/FTD, FTD	Autophagy; protein aggregation; axonal transport defects; proteasome impairment	[248,249]
*ALS2*	Alsin	2q33.1	ALS	Intracellular trafficking	[271,272]
*SQSTM1/p62*	Sequestome-1	5q35.3	ALS, ALS/FTD	Autophagy; proteasome impairment; protein aggregation	[178]
*FUS*	Fused in sarcoma	16p11.2	ALS, ALS/FTD	RNA processing; Protein aggregation	[297,298]
*TARDBP*	TDP-43	1p36.22	ALS, ALS/FTD	RNA processing; Protein aggregation	[121,122]
*SIGMAR1*	Sigma non-opioid intracellular receptor 1	9p13.3	ALS, ALS/FTD, FTD	Intracellular trafficking; proteasome impairment	[321,322]
*OPTN*	Optineurin	10p13	ALS	Autophagy; protein aggregation; Vesicular trafficking	[191]
*FIG4*	Polyphosphoinositide phosphatase	6q21	ALS	Intracellular trafficking	[112]
*UBQLN2*	Ubiquilin 2	Xp11.21	ALS, ALS/FTD	Autophagy; protein aggregation; proteasome impairment	[202]
*VCP/p97*	Valosin-containing protein	9p13.3	ALS, ALS/FTD, FTD	Autophagy; proteasome impairment; ER dysfunction; Vesicular trafficking;	[149]
*DCTN1*	Dynactin 1	2p13	ALS, ALS/FTD	axonal transport defects	[458]
*VAPB*	Vesicle-associated membrane protein	20q13.32	ALS	Intracellular trafficking; proteasome impairment	[343]
*TUBA4A*	Tubulin α4A chain	2q35	ALS, ALS/FTD	axonal transport defects	[473,474]
*SOD1*	Cu-Zn superoxide dismutase	21q22.11	ALS	Protein aggregation; proteasome impairment; axonal transport defects	[305]
*KIF5A*	Kinesin heavy chain isoform 5A	12q13.3	ALS	Intracellular trafficking; axonal transport defects	[446,447]
*GRN*	Granulin	17q21.31	FTD	Lysosomal function	[374,375]
*CHMP2B*	Charged multivesicular body protein 2b	3p11.2	ALS, ALS/FTD, FTD	Protein aggregation; autophagy	[364,366]
*TMEM106B*	Transmembrane protein 106B	7p21.3	FTD	Lysosomal function; axonal transport	[401]
*CCNF*	Cyclin F	16p13.3	ALS, ALS/FTD, FTD	Autophagy; protein aggregation	[355]
*SPG11*	Spatacsin	15q21.1	ALS	Vesicular trafficking	[478]
*PFN1*	Profilin 1	17p13.2	ALS, ALS/FTD, FTD	Membrane trafficking	[213,418-420]

^a^While more pathways can be/are affected by the ALS/FTD-related gene variants, only those directly linked to the autophagic or proteostasis pathways are mentioned.

## The impact of ALS/FTD-linked gene mutations on the autophagy and endolysosomal pathway

### TBK1

TBK1 (TANK binding kinase 1) is a serine/threonine kinase highly expressed in neuronal cells known to phosphorylate and thereby enhance the binding affinity of several (selective) autophagy receptors including OPTN, NDP52 (nuclear domain 10 protein 52) and p62/SQSTM1 to both LC3-II and their respective autophagic cargoes [123-129]. As a result, TBK1 is not only a key regulator of macroautophagy but also influences the efficiency of several selective autophagy pathways such as mitophagy and xenophagy (selective autophagy of bacterial pathogens) [123-129]. Despite originally associated with neuroinflammatory diseases, nearly 100 distinct mutations in *TBK1* were linked to ALS and FTD in 2015 and actually are mostly seen in patients with ALS/FTD [4,130-132]. The pathomechanism underlying *TBK1*-associated ALS/FTD most likely results from TBK1 loss-of-function as most *TBK1* mutations are either deletions that cause haploinsufficiency or nonsense and frameshift mutations that either negatively impact its function and kinase activity or result in nonsense-mediated mRNA decay and reduced expression of the kinase [130,131,133-135]. While TBK1 was found to be involved in the autophagosome maturation process [124], ALS/FTD-linked *TBK1* mutations seem to have the greatest impact on the OPTN-TBK1 interaction. As such, TBK1 mutants had a clear disruption in their interaction with OPTN, thereby reducing the recruitment of the autophagy receptor and LC3B to damaged mitochondria, impairing the mitophagy process and leading to the accumulation of dysfunctional mitochondria [125,128-130,136]. In fact, an elaborate study by Harding and colleagues that investigated the impact of 10 ALS/FTD-linked mutations in *TBK1* on mitophagy revealed that mutations that abolish either TBK1 dimerisation or reduce its kinase activity did not have a profound impact on mitophagy, while TBK1 variants that impacted both had a stronger disruptive impact on mitophagy [136]. Moreover, detailed analysis of the ALS/FTD-linked p.E696K *TBK1* missense variant that also leads to a selective loss of OPTN binding revealed neuronal autophagolysosomal dysfunction and an accumulation of damaged lysosomes [137]. In addition, TBK1 has been linked to C9orf72 as it was found to phosphorylate SMCR8, the key binding partner of C9orf72, thereby activating the latter protein and promoting autophagy initiation [138]. In fact, TBK1 knockdown in neuronal cells leads to the accumulation of p62 inclusions in a SMCR8-dependent manner as phosphomimetic SMCR8 was able to reverse this phenotype, underscoring the importance of this interaction [138]. Next to its role in (selective) autophagy, TBK1 recently emerged as a key regulator of the endolysosomal pathway as well. To start, TBK1 can phosphorylate Rab7, preventing its endolysosomal translocation and inhibiting late endosomal maturation [139,140]. Moreover, a recent study validated the role of TBK1 in endosome maturation and found that loss of TBK1 activity led to TDP-43 proteinopathy [108]. Next, TBK1 was found to control the trafficking of specific cargo and membrane proteins to MVBs for their degradation [141]. Finally, TBK1 is able to recognise aberrant endolysosomal organelles and target them to the autophagy (lysophagy) pathway which is crucial for long-term integrity of the endolysosomal system [142,143]. The toxic role of *TBK1* mutations seems smaller in rodents than in humans where they are rather classified as a risk factor for ALS/FTD as additional hits are needed to induce full-blown ALS/FTD pathology in mice [106,134,135].

### VCP

VCP (valosin-containing protein), also known as p97, is an AAA+ ATPase that is able to regulate a diverse range of cellular processes, including DNA damage repair, membrane dynamics, but mainly cellular proteostasis. It does the latter by regulating the autophagy pathway, reticulum–associated protein degradation (ERAD) and proteasomal degradation [144,145]. Disease-associated mutations in the *VCP* gene occur among the entire length of the protein and are found to cause loss-of-function of the ATPase [146]. *VCP* mutations are linked to numerous NDs including the multisystem proteinopathy called inclusion body myopathy associated with Paget disease of bone (PDB) and FTD (IBMPDD) [147], Charcot Marie Tooth disease type 2 (CMT2) [148] and ALS/FTD [146,149,150]. Despite presenting with distinct phenotypes, histopathological analyses of VCP patients and transgenic mice show a consistent pathology consisting of inclusions positive for ubiquitin, TDP-43 and p62. This is accompanied by ER-stress, damaged and enlarged (endo)lysosomes and an accumulation of autophagosomes marked by increased levels of p62 and LC3 reinforcing the major role VCP in the regulation of autophagy and proteostasis [147,149,151-155]. Interestingly, either knockdown or pharmacological inactivation of VCP was found to negatively affect MVB formation and autophagosome maturation, leading to a dysfunction of endolysosome-mediated protein degradation [153,155-157]. In addition, VCP interacts with EEA1 and clathrin and is able to regulate endosomal size and is thought to influence endocytosis, although no recent data support the latter [157,158]. Moreover, VCP mutants have been found to disrupt the autophagy-dependent removal of stress granules [159]. In fact, most research report a role for VCP mutations in the disruption of autophagy initiation, autophagosome maturation, autophagosome-lysosome fusion, mitophagy and lysophagy ultimately leading to the accumulation of defective mitochondria, autophagosomes and lysosomes and a disruption of the autophagic flux [153-155,160-162]. Finally, a recent study found that *VCP* mutations induce pathological accumulation of enlarged endolysosomes, damaged lysosomes and ER stress in iPSC-derived cortical neurons [163]. Interestingly, the researchers showed that this phenotype was, at least in part, caused by a pathogenic increase of 4 R tau isoforms, linking VCP to the tau protein which is the major constituent of aggregates in ~ 50% of FTD patients [2,163].

### SQSTM1/p62

SQSTM1/p62 (sequestome 1) is a multifunctional adaptor protein that is abundantly expressed in spinal cord MNs [164,165] and involved in many signalling pathways such as inflammation, oxidative stress, apoptosis, regulation of the ubiquitin-proteasome system and (selective) autophagy (reviewed in [166] and [167]). p62 is the first discovered autophagy adaptor (or receptor) protein that is able to recognise and bind ubiquitinated cargo via its ubiquitin-associated (UBA) domain and deliver them to autophagosomes through interaction with LC3 mediated by its LC3‐interacting region (LIR) for subsequent degradation [81,166-168]. In fact, since p62 itself is degraded during this process it is often used an indicator of autophagic flux [169]. Besides its well-studied role as cargo of ubiquitinated proteins, p62 also participates in aggrephagy, xenophagy, mitophagy and the selective autophagy of stress granules [126,170-172]. Activation of p62 occurs upon proteotoxic (or oxidative) stress and involves phosphorylation by ULK1, CK2 (Casein kinase 2) and/or TBK1, a process that increases its affinity for ubiquitinated cargo and enhances their removal [124,126,172-174]. In addition, p62 can also be activated by ubiquitination, which enhances it binding to and hence the removal of polyubiquitinated cargo [175,176]. *Post-mortem* tissue analysis revealed inclusions immunopositive for p62, that often also stained positive for ubiquitin and/or TDP-43, in both ALS and FTD patients [17-19,177]. Similar to VCP, mutations in p62 can either lead to distinct clinical phenotypes or result in a multisystem proteinopathy with coexisting signs of ALS/FTD [177-179], PDB [180] and inclusion body myositis (IBM) [181]. Most studied ALS/FTD-linked mutations in p62 are located in the promotor region and result in reduced p62 protein expression [182] or impair the recognition of ubiquitin or LC3 by p62 and thus impact cargo delivery to the autophagosome, always leading to a loss-of-function [168,177,183]. Indeed, knockdown of the p62 ortholog in zebrafish results in an ALS-like phenotype with locomotor defects, MN axon pathology and autophagy impairment [184]. Moreover, mTOR inhibition by administration of rapamycin or overexpression of wild-type p62 could ameliorate this phenotype while expression of the ALS/FTD-associated P392L p62 mutant could not [184]. Similarly, overexpression of wild-type p62 could attenuate RNA toxicity in a zebrafish model for *C9orf72*-ALS/FTD [185]. Moreover, p62 also interacts and exacerbates pathology associated with other ALS/FTD-linked disease proteins including TDP-43 and SOD1 [104,186]. Intriguingly, using the mutant SOD1^H46R^-expressing ALS mouse model to evaluate the impact of p62 knockout [186] or overexpression [187], interesting and seemingly conflicting results were obtained. Knockout of SQSTM1/p62 intuitively exacerbated disease progression by increasing the levels of insoluble SOD1 and accelerating the neuronal accumulation of ubiquitin-positive aggregates [186]. However, overexpression of SQSTM1/p62 also accelerated disease onset and shortened lifespan in this SOD1^H46R^ ALS mouse model. Interestingly, while insoluble p62 aggregates and polyubiquitinated protein where also significantly increased in the spinal cord of SOD1^H46R^ mice overexpressing SQSTM1/p62, these protein inclusions were mainly present in glial cells (astrocytes and/or microglia) rather than in neuronal cells [187]. These studies underscore the complexity of autophagy-associated genes in the pathogenesis of ALS and FTD and might even imply some time and context-dependent roles of autophagy during disease progression. In fact, using the established SOD1^G93A^ ALS mouse model, in combination with a MN-specific conditional knockout of Atg7, an important autophagy gene, distinct roles for neuronal autophagy were highlighted during disease progression [165]. Inhibition of autophagy in MNs initially accelerated neuromuscular junction loss and the onset of motor symptoms, but surprisingly, extended lifespan. Detailed analysis of these mice revealed that inhibition of autophagy did not rescue MN degeneration, but rather acts in a non-cell-autonomous manner as it silenced the overall glia-mediated inflammatory response and reduced activation of the c-Jun transcription factor in interneurons [165]. This study in particular highlights the importance of taking into account disease progression when targeting autophagy as it may play distinct roles, and its modulation may have drastically different results, depending on the relative disease progression status.

### OPTN

Although mutations in the *OPTN* (optineurin) gene were originally found to cause a specific type of glaucoma [188,189], they were later identified as a rare cause of both FTD [190] as well as ALS [189,191]. Similar to p62, OPTN is a multifunctional scaffolding protein abundantly expressed in the brain [188] and involved in various cellular function, including NF-κB activation and immune signalling, exocytosis, vesicular trafficking and autophagy [192,193]. However, OPTN is best known for its role as an autophagy (mitophagy) receptor that links ubiquitinated cargo, especially mitochondria, but also aggregates and pathogens, to LC3-positive autophagosomes via its UBA and LIR domains respectively [79,81,82,123,125,194,195] or in an ubiquitin-independent manner [195,196]. Moreover, OPTN interacts with myosin IV promoting autophagosome maturation and their fusion with lysosomes [197,198]. *OPTN* mutations are though to act in a loss-of-function manner as most disease-associated variants result in decreased OPTN protein levels or impair the function of the UBA domain of OPTN [190,191]. In line with this, *OPTN* deletion led to the accumulation of aggregates [196] and expression of various OPTN mutants causes a motor phenotype that was also characterised by a disruption in the autophagic flux [199], defects in autophagosome maturation [198] mitophagy, xenophagy and aggrephagy [123,196] and impaired phagophore formation [199,200]. Finally, an interactome study comparing wild-type to ALS-associated E478G OPTN protein revealed a dramatic reduction in the amount of interaction partner of OPTN, especially with protein involved in ER transport and proteostasis [201].

### UBQLN2

UBQLN2 (ubiquilin-2) is one of four mammalian ubiquilin proteins and functions as a proteasomal adaptor protein capable of binding ubiquitinated cargoes through its UBA domain and, in concert with HSP70, shuttling them to the proteasome for degradation [202-205]. UBQLN2 is also implicated in the autophagy pathway although its role there is less well characterised and thought to be more complex [206,207]. First, UBQLN2 may act as an autophagy receptor and target ubiquitinated targets to LC3-positive autophagosomes [208,209]. Second, UBQLN2 interacts with LC3 and OPTN to promote autophagy induction and autophagosome formation [208-210]. Third, UBQLN2 was found to regulate mTORC1 activity with loss of UBQLN2 levels or activity resulting in increased autophagy induction [211]. Fourth, UBQLN2 negatively regulates CMA [206]. Last, ubiquilins, including UBQLN2, were recently found to be involved in the regulation of vacuolar ATPase levels as two independent groups uncovered two non-mutually exclusive mechanisms by which UBQLN2 interacts with the v-ATPase complex and promotes its assembly and function [211,212]. It was therefore not surprising that disruption of UBQLN2 function lead to impairment of lysosomal acidification and a reduction of the autophagic flux. Variants in *UBQLN2* were found to be causative for X-linked forms of ALS and ALS/FTD [202,203,213,214], and results in the presence of UBQLN2 in neuronal cytoplasmic aggregates positive for ubiquitin, p62 and TDP-43 [202,215]. Interestingly, UBQLN2 directly interacts with TDP-43 and it known to regulate its levels through the autophagic pathway [216]. In fact, UBQLN2 overexpression is able to reduce TDP-43 aggregation providing additional evidence for a causative role for autophagy dysfunction in the development of TDP-43 proteinopathies [216]. Coinciding with its role in lysosomal function, both rodent and iPSC-derived neuronal models of UBQLN2-ALS reported enlarged LAMP1-positive vesicles that colocalized with UBQLN2 aggregates and the endosomal protein Rab5 was found to be a modifier of this pathology [217,218]. Surprisingly, UBQLN2 knockout models in mice or rats only induced mild age-dependent motor defects [219], while either increased expression of WT UBQLN2 protein or transgenic expression of ALS/FTD mutant UBQLN2 resulted in varying levels of neurodegeneration and motor defects together with the accumulation of ubiquitinated aggregates positive for p62, LC3, TDP-43 and UBQLN2 itself [215,217,220-223]. Of note, transgenic mice expressing intermediate levels of P506T-UBQLN2, which causes an aggressive early-onset form of ALS/FTD, did present with alterations in ubiquitin-dependent protein homoeostasis and the formation of widespread neuronal inclusions but lacked clear signs of neurodegeneration [224]. Indeed, a recent cellular study that compared 5 *UBQLN2* mutations found a huge difference in aggregation propensity and neurotoxicity between all pathogenic variants [225]. Neuronal expression of the ALS-linked P497H UBQLN2 variant was found to exacerbate TDP-43 pathology in a TDP-43 mutant mouse model [226], suggesting that additional hits might be needed to induce ALS/FTD and that both loss- and gain-of-function mechanisms are at play in UBQLN2-ALS/FTD [224].

### C9orf72

*C9orf72* transcripts and protein are found in most tissues, but the highest levels are found in the CNS (brain and spinal cord) and in the immune system [227-231]. Interestingly, several studies revealed co-localisation of C9orf72 with components of the endolysosomal pathway including early/late endosomes, lysosomes, autophagosomes and phagolysosomes [138,232-240]. In neuronal cells, co-localisation with synapse-specific markers was shown and proteome analysis of the synaptosome confirmed the apparent synaptic localisation of C9orf72 [232,234,241,242]. This is consistent with the proposed physiological role of C9orf72 which is found to be involved in multiple pathways including stress granule homoeostasis, actin dynamics and axonal growth, nucleocytoplasmic transport and membrane trafficking events crucial for the autophagic and endolysosomal systems [66]. In fact, the C9orf72 protein is part of a bigger complex consisting C9orf72, SMCR8 (SMCR8-C9orf72 complex subunit), and WDR41 (WD repeat domain 41) that interacts with a variety of Rab GTPases and, although it is predicted to function as a GEF (guanine exchange factor) for these Rab proteins, recent evidence rather supports a function as GAP (GTPase-activating protein) for the C9orf72 complex [138,239,243-247]. Hence, by activating RAB GTPases, C9orf72 is implicated in the regulation of membrane trafficking events, including endocytosis, phagocytosis and multiple parts of the autophagy and endolysome pathway. In 2011, independent research groups made a groundbreaking discovery and were able to ascribe over 30-55% of fALS, 20-25% of fFTD and actually the majority of familial ALS/FTD cases to “GGGGCC” hexanucleotide repeat expansions (HREs) in the 5’ non-coding sequence of the *C9orf72* gene, making it the most common genetic cause of ALS/FTD [248-250]. Healthy, unaffected individuals carry between 2-30 of these repeats, while affected *C9orf72* patients present with hundreds up to thousands of repeats [248,249,251]. Three distinct but not mutually exclusive disease mechanisms have been proposed to underlie *C9orf72* HRE-associated pathology [252,253]. These include C9orf72 protein haploinsufficiency triggered by reduced transcription of the *C9orf72* gene alongside two toxic gain-of-function mechanisms being RNA-binding protein sequestration by *C9orf72* HRE RNA-containing RNA foci and the production of five different dipeptide repeat proteins (DPRs) produced by repeat-associated non-AUG (RAN) translation of the HRE [252-254]. Multiple groups found that, when expressed in cells, flies or mice, the HRE results in the accumulation and enlargement of endolysosomes and a decreased nuclear TFEB signal [255,256]. Intriguingly, poly(GA), one of the five DPRs produced from the HRE was found to drive pathological enlargement of endosomes, TDP-43 pathology, autophagic defects and neuronal loss in mice through sequestration of TBK1, a process that is even worsened if additional mutations in *TBK1* were introduced [106,257]. In addition, a recent study confirmed the detrimental effects of the *C9orf72* HRE mutation on endolysosomal health and TBK1 function in iPSC-derived MNs [258]. In fact, this study revealed lysosomal transport defects, disrupted lysosomal homoeostasis, inhibition of the autophagic flux and accumulation of p62 in *C9orf72-*ALS patient neurons [258]. However, while C9orf72 loss-of-function did not impact endolysosomal morphology or function, an impairment in the maturation of early endosomes upon C9orf72 loss was seen [258]. Moreover, interactome screening of all five DPRs revealed sequestration and functional impairment of VCP by poly(GA), similar to TBK1, linking yet another endolysosome-related protein to C9orf72 ALS/FTD [259]. Several groups have reported defects in autophagic flux, increased sensitivity to autophagy inhibitors and (glutamate-induced) MN degeneration in *C9orf72* iPSC-MNs [235,258,260-262]. Although toxic gain-of-function mechanisms clearly impact autophagy as mentioned above, most research so far has focused on the physiological role of C9orf72 in autophagy and its dysregulation in ALS. This uncovered a role for the C9orf72 protein complex in the regulation of ULK1 [119,138,263] and mTORC1 [237,264,265] reviewed in detail elsewhere [66]. Moreover, dysregulation of the interaction between C9orf72 and its several Rab-interactors not only causes defects in autophagosome biogenesis, but also impact axonal transport of autophagic vesicles and autophagosome-lysosome fusion events [138,233,235,238,239,243-246,263,266].

While the majority of the field attributes a major role for toxic gain-of-function mechanisms in *C9orf72* ALS/FTD pathogenesis, mouse models revealed that C9orf72 deficiency exacerbates disease [267,268]. However, *C9orf72* knockout mice presented with immune system dysregulations marked by splenomegaly, glial dysregulation and overproduction of inflammatory cytokines rather than neurodegeneration [229-231]. This discrepancy can be explained by the fact that C9orf72 mRNA levels vary a lot between the different cell populations present in the brain and spinal cord and C9orf72 is predominantly expressed in myeloid cells such as microglia and macrophages [227,228]. A recent single nuclei profiling study showed that C9orf72 was reduced mainly in microglia rather that in neurons [227]. In addition, *C9orf72* knockout iPSC-derived MNs failed to show overt signs of neurodegeneration or autophagy dysfunction pointing towards a minor role of C9orf72 in neurons [258]. These results are further supported by both patient iPSC-derived microglia with C9orf72 haploinsufficiency [269] and *C9orf72* knockout iPSC-derived microglia cells [270] which showed impairments of phagocytosis and a hyper-active immune response.

### ALS2

Recessive homozygous missense mutations in the *ALS2* gene, which encodes for Alsin, cause a rare juvenile form of ALS characterised by loss-of-function of the native protein [271,272]. Similar to C9orf72, alsin is associated with the endosomal network and the autophagy pathway by its role as a GEF interacting with Rab proteins, especially the small GTPase Rab5 [273,274]. Rab5 is a key player in endosome dynamics and autophagosome formation and ALS-linked mutations in *ALS2* were indeed found to impair endosomal maturation, negatively impact the formation of autophagosomes and amphisomes and decrease the autophagic flux [273-277]. Concordantly, loss of alsin in primary neurons resulted in decreased motility and impaired maturation of Rab5-positive endosomes and a defect in the Rab5-dependent early endosome fusion process resulting in the accumulation of pathologically enlarged EEA1-positive early endosomes [278-280]. Although ALS2 has no GEF-activity towards Rab17, their physical interaction promotes Rab11-mediated recycling endosomes to transform into EEA1-positive early endosomes, a process that is probably also impacted by ALS mutations in *ALS2* [281]. Simultaneous deletion of *ALS2* and *SQSTM1* in a model for *SOD1*-ALS revealed distinct but additive effects of both genes on the disease phenotype and pathology, further highlighting the role of autophagy and the endolysosomal system in ALS [186].

### TARDBP

*TARDBP* encodes for TDP-43, a RNA/DNA-binding protein which is mislocalized from nucleus to cytoplasm where it aggregates in approximately 97% and 40% of ALS and FTD cases respectively [3,4,282]. Interestingly, the link between TDP-43 and autophagy is threefold. First, autophagy is crucial in maintaining TDP-43 protein homoeostasis as aggregated and misfolded TDP-43 as well as toxic aggregation-prone C-terminal fragments of the protein are degraded by the autophagic pathway, while soluble TDP-43 species are primarily degraded by the UPS and chaperone mediated autophagy (CMA) [94,283-285]. Second, activation or restoration of the autophagy-lysosome pathway by multiple different methods in a variety of model systems has proven to ameliorate TDP-43-related neurodegeneration [94,286-289]. Third, both toxic gain-of-function mechanisms by aggregated, misfolded or C-terminal fragments of TDP-43 and loss-of-function mechanisms of TDP-43 result in dysregulation of the autophagic pathway [100,290-293]. In fact, TDP-43 transcriptionally regulates ATG7 and ATG4B mRNAs and its aggregation results in reduced mRNA levels of ATG7 which impairs autophagy initiation [292,294]. Similarly, TDP-43 also regulates RPTOR (Regulatory‐Associated Protein of mTOR) and DCTN1 (Dynactin 1) and while loss-of-function of TDP-43 was shown to result in increased autophagosome and lysosome biogenesis in a TFEB-dependent manner by inhibiting mTOR activity, it also reduced dynactin levels, a crucial component of the autophagosome-lysosome fusion machinery [290,293]. This inevitability leads to the build-up of autophagosomes and disruption of the autophagic flux [295]. On another note, TDP-43 loss-of-function was also found to influence endosomal trafficking as knockdown of TDP-43 decreases the number and mobility of (Rab11-positive) recycling endosomes by upregulation of VPS4B, a key component of the ESCRT complex [296]. These observations underscore the multifaceted role of TDP-43 in the endolysosomal pathway and has led to the hypothesis of a negative regulatory feedback loop underlying ALS/FTD as TDP-43 sequestration into aggregates leads to reduced functional TDP-43 levels, in turn leading to defective lysosomal degradation pathways, which in turn will aggravate the TDP-43 aggregation, eventually culminating in neurodegeneration [295].

### FUS

Similar to TDP-43, FUS is another RNA/DNA-binding protein which is mislocalized and aggregated in both familial ALS (1%) and FTD (5-10%) cases [3,4,297-299]. Adding to this similarity, the endolysosomal system is pivotal for the clearance of aggregated and misfolded FUS species and activation of the autophagy pathway by a variety of methods successfully lowered FUS inclusions and mitigated neurotoxicity [300-302]. Moreover, both loss-of-function of FUS and toxic gain-of-function by mutants FUS are able to directly influence autophagy. Upon depletion of FUS in neuronal like N2A cells, mRNA levels of several key factors in the autophagy initiation process are lowered including *RB1CC, FIP200, ATG12* and *ATG16L1* [303]. Expression of mutant FUS in neuronal cell lines negatively affected autophagosome formation and lead to the accumulation of p62 and a decrease in autophagic flux [301]. Intriguingly, these defects could be reversed by overexpression of Rab1, suggesting a potential interaction between mutant FUS and Rab1 function [301]. Finally, general overexpression of wild-type FUS in mice or N2A cells was not only associated with dysregulation of RNA metabolism, but also impaired autophagosome formation ad maturation [304].

### SOD1

Mutations in the *SOD1* (Cu/Zn superoxide dismutase 1) gene that encodes for an antioxidant enzyme were the first to be found causative for ALS [305]. Numerous studies have reported that stimulating autophagy or mitophagy can degrade mutant and/or aggregated SOD1 and slow down disease progression [306-312] while inhibition of autophagy aggravates neurodegeneration in *SOD1-*ALS models [165]. Moreover, *SOD1* transgenic mice heterozygous for the autophagy regulator BECN1 showed an increase in survival while having increased p62 and reduced LC3-II levels, which is found to be mediated by an abnormal interaction of mutant SOD1 with the BECN1-BCL2L1 complex that may impact autophagy stimulation [313]. However, caution is warranted when evaluating these results in the context of *SOD1*-ALS as treatment of mutant *SOD1* mice with the autophagy inducing drug rilmenidine effectively promoted the autophagy-dependent removal of mutant SOD1 protein but worsened MN degeneration, SOD1 aggregation and disease progression [314]. Moreover, while treatment of *SOD1*-ALS mice with rapamycin successfully delayed symptom onset, overall disease progression was worsened, and survival of rapamycin-treated mice was shortened when compared to untreated *SOD1*-ALS mice [315]. This can be explained by the possible off-target and non-cell autonomous effects of autophagy activation or inhibition [165]. Overall, the impact of *SOD1* mutations on autophagy is complicated as on the one hand both mRNA and protein levels of the autophagy regulating protein TFEB were decreased in *SOD1* mice [316] and mutant SOD1 (aggregates) are able to sequester essential components of the autophagy pathway such as BECN1 [313] and optineurin [317] while on the other hand, an increase of autophagy was found in *SOD1*-ALS mice [318,319]. Finally, researchers recently discovered that SOD1 is actively being secreted from cells by a process called secretory autophagy where autophagosomal organelles containing SOD1 protein fuse with secretory lysosomal-related organelles and undergo exocytosis [320]. Deletion of Plekhg5, an important player in this pathway, in *SOD1*-ALS mice led to a reduced secretion of toxic SOD1 protein species, accelerated disease onset but prolonged survival due to an attenuation of microglial activation [320]. This again highlights the importance of other cell types, especially glial cells, in neurodegenerative diseases and their treatment.

### SIGMAR1

Recessive mutations in the *SIGMAR1* gene encoding the sigma receptor 1 (SigR1) protein are a rare cause of (juvenile) ALS/FTD [321,322]. SigR1 is an ER chaperone protein involved in a variety of biological processes (reviewed in [323]) but expression of ALS/FTD-linked SIGMAR1 in cellular and mouse models negatively affects autophagy most likely due to a loss-of-function mechanism as SigR1 activation or overexpression can rescue these defects [324,325].Recent studies revealed that either expression of ALSD/FTD SigR1 mutants or ablation of endogenous SigR1 caused impaired endosomal trafficking and autophagic flux defects mediated by impairments in the autophagosome-lysosome fusion process [326-328]. Moreover, a recent study unveiled a novel function for SigR1 in the regulation of autophagy as the molecular chaperone was found to interact with POM121 (an important nucleoporin linked to C9orf72 ALS/FTD [329]) and regulate TFEB nucleocytoplasmic transport [330].

### FIG4

Although mutations in the gene encoding FIG4 (Factor-induced gene 4) were first identified as a cause of Charcot-Marie-Tooth (CMT), a peripheral neuropathy, they were later also found to be a rare (1-3%) cause of ALS, especially in central European cohorts [112,331,332]. FIG4 is a phosphoinositide phosphatase crucial for the regulation of PI [3,5] P2 (phosphatidylinositol-3,5-bisphosphate) levels, a key signalling lipid in the autophagy and endolysosomal pathway, by mediating its conversion to PI [3] P (phosphatidylinositol-3-phosphate) [333,334]. In fact, PI [3,5] P2 is important for retrograde endosomal trafficking, endolysosomal protein degradation and endosomal maturation [333-336]. Regulation of PI [3,5] P2 levels is a complex issue since the kinase PIKfyve, which has a function opposite to FIG4 (thus converting PI3P to PI [3,5]P2), is part of a bigger complex together with FIG4 present mainly on late endosomes where they can regulate each other activity [333,337,338]. Thus, given the known interaction with PIKfyve, *FIG4* mutations could in theory either increase or decrease PI [3,5] P2 levels. Indeed, while knockdown of *FIG4* dramatically increased cellular levels of PI [3,5] P2 [333], expression of FIG4 mutants in mice actually led to a reduction of PI [3,5] P2 and had negative effects on survival [331]. More specifically, both the neuron-specific and constitutive knockout of *FIG4* in mice led to a neurodegenerative phenotype and the accumulation of p62, LC3-II and LAMP1/2 in astrocytes and neurons [334,339]. Interestingly, both *Fig4* null flies [340] and *Fig4* null mice [341] display endosomal and lysosomal phenotypes that can be rescued by the expression of either wild-type, catalytically inactive or ALS-linked *FIG4* transgenes with mutations predicted to inactivate the kinase function of FIG4. Therefore, the general consensus on the mechanistic basis underlying FIG4-ALS is independent of the phosphatase activity of FIG4 but rather based on loss-of-function and the inability of FIG4 mutants to stabilise the PIKfyve complex.

### VAPB

VAPB (vesicle-associated membrane protein-associated protein B) is an ER protein that acts as a tether between the ER and other organelles including the Golgi complex and vesicles of the endolysosomal system [342]. Mutations in VAPB represent a rare cause of ALS [343,344] and are mainly thought to achieve this toxicity through loss of VAPB’s endogenous function [345-347]. In fact, reduced levels of VAPB have been observed fibroblasts and iPSC-derived motor neurons from *VAPB*-ALS patients when compared to their non-carrier siblings [345] and in spinal cord MNs of sALS patients when compared to healthy controls [348,349]. VAPB interacts with many proteins that modulate autophagosome biogenesis (reviewed in [350]) but can also trigger the activation of mitophagy [351] and ER-phagy [352] by interacting with the mitochondrial PTPIP51 (tyrosine phosphatase interacting protein 51) protein or the ER-phagy receptor CALCOCO1 (calcium binding and coiled-coil domain 1) respectively. In line with this, analysis of spinal cord tissue from a mutant *VAPB*-ALS mouse model revealed elevated p62 and LC3 levels and an alteration of the autophagic flux [346,353]. More detailed analysis of the autophagic flux in *VAPB*-ALS patient fibroblasts revealed defects in both the initiation of autophagy and the autophagosome-lysosome fusion process resulting in an accumulation of autophagosomes [353]. VAPB also regulates PI4P (phosphatidylinositol-4-phosphate) formation on endosomes and loss of VAPB results elevated PI4P levels in the Golgi complex and an accumulation of endosomes derived from this organelle. When an excess of these Golgi-derived endosomes fuse with lysosomes, they lead to dysfunctional lysosomes with an aberrant acidity, shape and content and hence influence both the endolysosomal and autophagy pathway [354].

### CCNF

Mutations in the *CCNF* gene, which encodes for Cyclin F, a component of the E3 ubiquitin-protein ligase complex, are a rare cause of familial and sporadic ALS and FTD [355,356]. Interestingly, cyclin F has a plethora of ALS/FTD-relevant interaction partners including VCP, TDP-43, FUS, p62, OPTN, SOD and TBK1 [357,358]. While the exact pathological mechanism of *CCNF* mutations in ALS/FTD remains debated, overexpression of the specific ALS/FTD-linked S621G CCNF mutation increased the interaction and led to the aberrant ubiquitylation of the autophagy receptor p62 and resulted in the impairment of autophagosome-lysosome fusion [359]. Recently, the mechanism behind the dysregulation of the cyclin F-p62 axis was uncovered as cyclin F-dependent ubiquitylation of p62 was found to regulate its ability to aggregate and form cytoplasmic foci and that expression of the ALS and FTD-lined S621G CCNF mutant causes aberrant ubiquitylation and increased aggregation of p62 [360].

### CHMP2B

CHMP2B (charged multivesicular body protein 2B) is part of the ESCRT-III complex which functions in membrane deformation and regulates MVB formation and maturation, autophagosome formation, autophagosome-lysosome fusion, endosomal sorting and endolysosomal trafficking [361-363]. Although missense mutations in *CHMP2B* were initially associated with FTD [364,365], they were later also found in a specific ALS subgroup with lower motor neuron predominance [366,367]. Transgenic mouse models expressing mutant CHMP2B develop pathology reminiscent of ALS and FTD that coincide with the enlargement of late endosomes, an accumulation of autophagosomes, defects in autophagy and a lysosomal storage pathology [114,368,369]. Interestingly, *CHMP2B* knockout mice did not present with neurodegenerative phenotypes implicating a predominant toxic gain-of-function mechanism in *CHMP2B*-associated ALS and FTD [368]. ALS or FTD-linked CHMP2B mutants have aberrant autoregulation which results in the prolonged engagement of the ESCRT-III complex with the MVB membrane causing defects in endosome/MVB-lysosome fusion events and general endolysosomal function that coincide with autophagosome accumulation and a reduction in the autophagic flux [363,365,367,370-372]. In addition, a recent screen in mutant *CHMP2B* Drosophila models discovered TBK1 and dynein, two factors involved in early endosome trafficking as modifiers of mutant CHMP2B toxicity [373], adding another layer of evidence for the involvement of both endolysosomal and autophagy dysregulation in the pathology of CHMP2B-ALS/FTD.

### GRN

Loss-of-function mutations in the *GRN* (granulin) gene cause neurodegeneration in a dose-dependent manner. Heterozygous GRN mutations result in haploinsufficiency of the PGRN (progranulin) protein and are associated with up to 10% of all FTD cases and around 25% of familial FTD worldwide [374-376]. Homozygous loss of PGRN on the other hand results in an even more severe ND and is associated with neuronal ceroid lipofuscinosis (NCL), a type of lysosomal storage disorder which directly links PGRN to lysosomal biology [377]. PGRN is a secreted glycoprotein and neurotrophic factor that can promote neuronal survival and enhance their outgrowth [378] and which, besides its role in the regulation of lysosomal function [379,380], has a plethora of other cellular functions ranging from angiogenesis, inflammation, phagocytosis, apoptosis, cell migration and metabolic regulation (reviewed in [381] and [382]). Downstream processing of progranulin in its effector granulin fragments mainly takes place in lysosomes and requires effective lysosome acidification [383]. Defects in PGRN processing could therefore be a sign of dysregulated endolysosomal acidification and function, but since PGRN itself can also mediate lysosome acidification and homoeostasis itself, for example by aiding the maturation cathepsin D, a lysosomal protease, it not known whether this is a cause or consequence of PGRN haploinsufficiency [384-386]. In fact, reduction in PGRN levels resulted in increased lysosomal biogenesis as marked by elevated levels of lysosomal gene expression and protein levels and both cellular models of *GRN*-FTD as well as heterozygous or homozygous *GRN* knockout mice present with an accumulation of lysosomes that are unable to mature or fuse properly and have an aberrant proteome [387-392]. The relation between PGRN deficiency and autophagy is more complex as the levels of PGRN itself are controlled by the autophagic pathway [393]. Consequently, complete loss or haploinsufficiency as well as overexpression of PGRN and has been found to impair autophagic flux, implying a complex interplay and tightly regulated balance between PGRN and the autophagy-lysosome system [393-395].

### TMEM106B

Although not being a direct genetic cause of ALS or FTD, genetic variants in the gene encoding TMEM106B (transmembrane protein 106B), a membrane protein especially present in neuronal and glial cells on (endo)lysosomes positive for Rab7, Rab9 and LAMP1 [396-400] and interactor of PGRN, are identified as risk factors for GRN-FTD and C9orf72-FTD [401-405]. These SNPs can either increase or decrease mRNA and protein levels of TMEM106, both of which affect lysosomal homoeostasis [399-402,405,406]. Overexpression of TMEM106B resulted in the activation of TFEB-dependent lysosomal biogenesis and these cells accumulated improperly acidified and enlarged (endo)lysosomes and autophagosomes, showed defects in MPR trafficking and an increase in the retrograde axonal transport of lysosomes [397-399,407-409]. Loss of TMEM106B in GRN or C9orf72 mouse models on the other hand yielded fewer and smaller lysosomes and caused autophagic stress, mainly due to problems with lysosomal acidification [408,410-413]. Interestingly, reducing C9orf72 protein levels was able to alleviate the lysosomal defects caused by TMEM106B overexpression in cells [409], while a reduction of TMEM106B could only partially rescue neuronal loss in a C9orf72 ALS/FTD mouse model [414] adding another layer to the complex relationship between *GRN, C9orf72* and *TMEM106B* in lysosomal health and neurodegeneration. Finally, as mentioned before, intracellular amyloid fibrils containing a C-terminal fragment of TMEM106B have recently been found in various NDs including ALS and FTD suggesting that TMEM106B dysregulation may be a common feature associated with neurodegeneration [115-117]. However, given the fact that these fibrils are also found in the brain of aged (non-demented) individuals they could also be linked to the general proteostasis decline that is inevitably associated with the process of ageing itself [415,416]. Therefore, more research is needed to understand the formation and (pathogenic) role of native TMEM106B protein and its fibrillar counterpart in health and disease.

### PFN1

PFN1 (profilin 1) is a nucleotide exchange factor that binds actin monomers and is vital for its polymerisation into actin filaments [417]. Mutations in *PFN* have been linked to rare familial forms of ALS and FTD [213,418-420]. Interestingly, PFN1 also binds to VCP [421], another ALS-related protein and regulator of autophagosome maturation, and PTEN [422] (phosphatase and tensin homolog), a phosphatidylinositol 3,4,5-trisphosphate 3-phosphatase that dephosphorylates PIP3 tot PIP2 thereby also influencing the autophagy pathway. This involvement with autophagy is strengthened by the fact that mutant PFN1 protein was found to form ubiquitin -and p62-positive aggregates in the cytoplasm of cells and mouse neurons expressing ALS-linked PFN1 mutants [418,423]. Moreover, these aggregates were found to sequester both LC3 and TDP-43 [418,423]. Although these studies suggest a toxic role for PFN1 aggregates, transgenic C71G-PFN1 mice developed a fast progressive ALS phenotype and presented with elevated ubiquitin and p62 levels that eventually formed aggregates but only after disease onset was already triggered [424]. Recently, two independent groups discovered a role for PFN1 in mitochondrial homoeostasis as knockout of the actin binding protein was found critical for mitochondrial function as resulted in the activation of mitophagy [425] while expression of the M114T PFN1 mutant deregulated Rab9-dependent mitophagy [426]. Moreover, iPSC-derived microglia harbouring C71G and M114T PFN1 mutants impaired vesicular degradation pathways and phagocytosis, at least in part due to increased binding with PI3P [427].

As mentioned previously, neurons have a specialised cell-type-specific regulation of the autophagy-lysosome pathway owing to their highly polarised nature [55-60]. Axonal transport is of specific importance as its dysfunction is observed in several NDs including ALS and FTD and is considered an early pathogenic feature [428-430]. Neuronal axonal transport is crucial for the proper functioning of the autophagy system as autophagosomes are formed distally at the synapses and require retrograde trafficking to the soma in combination with anterograde trafficking of (endo)lysosomes in order to fuse and mature [60,430]. Preclinical data obtained in murine or iPSC models of the major familial ALS subtypes revealed axonal transport defects due to pathogenic variants in the genes encoding for *FUS* [431-433], *SOD1* [434-436], *TARDBP* [437-439] and *C9orf72* [440-443]. Moreover, mutations in components of the axonal transport machinery including *KIF5A, DCTN1, TUBA4A* and *SPG11* have been directly linked to ALS/FTD.

### KIF5A

Mutations in the gene encoding KIF5A (kinesin 5A), a neuron-specific anterograde molecular motor protein, are causative for hereditary spastic paraplegia (HSP) [444], Charcot-Marie-Tooth disease type 2 (CMT2) [445] and ALS [446,447]. Interestingly, while pathogenic variants associated with ALS are located in the tail (cargo-binding)- domain of the molecular motor, CMT and SPG-linked mutations mainly reside in the motor (microtubule-binding) domain of KIF5A [448]. Lysosomes and mitochondria are among the most common cargos of KIF5A, linking it to general autophagy and mitophagy respectively [449,450]. Indeed, deficiency of KIF5A results in axonal transport defects that impair the autophagic flux and provide evidence for a loss-of-function toxicity mechanism in *KIF5A*-ALS [449]. However, the recently discovered KIF5A variants that cause exon 27 skipping (KIF5A ΔExon27) also suggest a toxic gain-of-function role as these mutations result in KIF5A proteins with altered C-terminal domains that are prone to form neurotoxic aggregates, while also having an increased microtubule processivity resulting in dysregulated axonal transport [450-452].

### DCTN1

DCTN1 (Dynactin subunit 1) is the largest component of the dynactin protein complex that interacts with the retrograde motor protein dynein in order to initiate and stimulate retrograde transport [453,454]. This is achieved by enhancing the processivity of its transport and by functioning as an adaptor protein to tether organelles (including endosomes, lysosomes and autophagosomes), RNA granules and other specific cargos to dynein [455,456]. Mutations in *DCTN1* have been linked to various TDP-43 proteinopathies such as Perry syndrome [457], lower motor neuron disease [458], ALS [459,460] and ALS/FTD [461]. Interestingly, all these *DCTN1*-linked NDs are characterised by TDP-43 pathology hinting towards a direct link between axonal transport and TDP-43 proteinopathies [462]. Although some ALS-causing *DCTN1* mutations did not significantly alter DCTN1 protein structure, most DCTN1 mutants disrupt the interaction of the protein with either dynein or microtubules resulting in toxic aggregates containing mutant DCTN1, its cargo and TDP-43 [463,464]. Furthermore, while heterozygous germline knock-out [465] or complete neuron-specific ablation [466] of *DCTN1* in mice only resulted in no or very mild MN degeneration, heterozygous knock-in of the G59S mutation lead to halving of DCTN1 protein levels accompanied with MN degeneration, astrogliosis, defective vesicular transport and the accumulation of LC3-II-positive DCTN1 aggregates [463,465,467]. Although the evidence stated above mainly points towards a gain-of-function mechanism in DCTN1-ALS, multiple studies reported that reducing or ablating the levels of the *DCTN1* orthologues in either *C.elegans* [468], *Drosophila* [469] or zebrafish [470] results in motor deficits and axonal pathology independent of DCTN1 aggregation. Finally, analysis of *post-mortem* tissue from sALS patients identified reduced levels of *DCTN1* mRNA in MNs [471] and lower DCTN1 protein levels in the motor cortex [472]. Thus, while the involvement of *DCTN1* mutations in ALS/FTD pathogenesis is clear, the question whether these mutations cause disease through gain -or loss-of-function remains a source of debate and requires further investigation.

### TUBA4A

*TUBA4A* (α-tubulin isoform 4a) encodes one of nine eukaryotic α-tubulin isotypes which heterodimerize with β-tubulin to form the microtubule network. Despite being ubiquitously expressed, TUBA4A is particularly enriched in the nervous system [473] and albeit rare, mutations in *TUBA4A* were found to be causal for familial cases of ALS and ALS/FTD [473,474]. Moreover, additional mutations in the *TUBA4A* gene were observed in sporadic patients [475] and a general decrease of TUBA4A protein levels have been described in ALS-affected brain regions [476,477]. Although inclusions containing mutant TUBA4A can be formed, these data mainly support a loss-of-function mechanism. Indeed, a recent study reported that knockdown of the *TUBA4A* orthologue in zebrafish induced an ALS phenotype with MN abnormalities and defects in motor behaviour [477]. The gross majority of *TUBA4A* mutations reside in the interaction domains with either β-tubulin and affect tubulin dimerisation, microtubule stability and dynamics or with the molecular motor proteins dynein and kinesin thereby impairing microtubule-based axonal transport [473-475]. In conclusion, most evidence linking autophagy to TUBA4A mutations involve the disruption of dynein-based retrograde transport of autophagosomes and concomitant impairment of autolysosome formation through a loss-of-function mechanism.

### SPG11

*SPG11* (spastic paraplegia 11) encodes for the spatacsin protein and although its function not being completely understood, is thought to control gene regulation, vesicular trafficking and axonal maintenance. Mutations in *SPG11* are linked to both hereditary spastic paraplegia (HSP) and juvenile ALS [478-480]. Loss of spatacsin function results in autophagy defects marked by a reduction in lysosome number and accumulation of autophagosomes and has an overall detrimental effect on lysosomal pathways due to reduced lysosomal reformation and biogenesis [481-483], dysfunctional anterograde axonal trafficking [484,485] and impaired clearance and hence the accumulation of cholesterol in lysosomes [486].

## Therapeutic strategies targeting autophagy for the treatment of ALS and FTD: challenges and limitations

**Figure 2.**
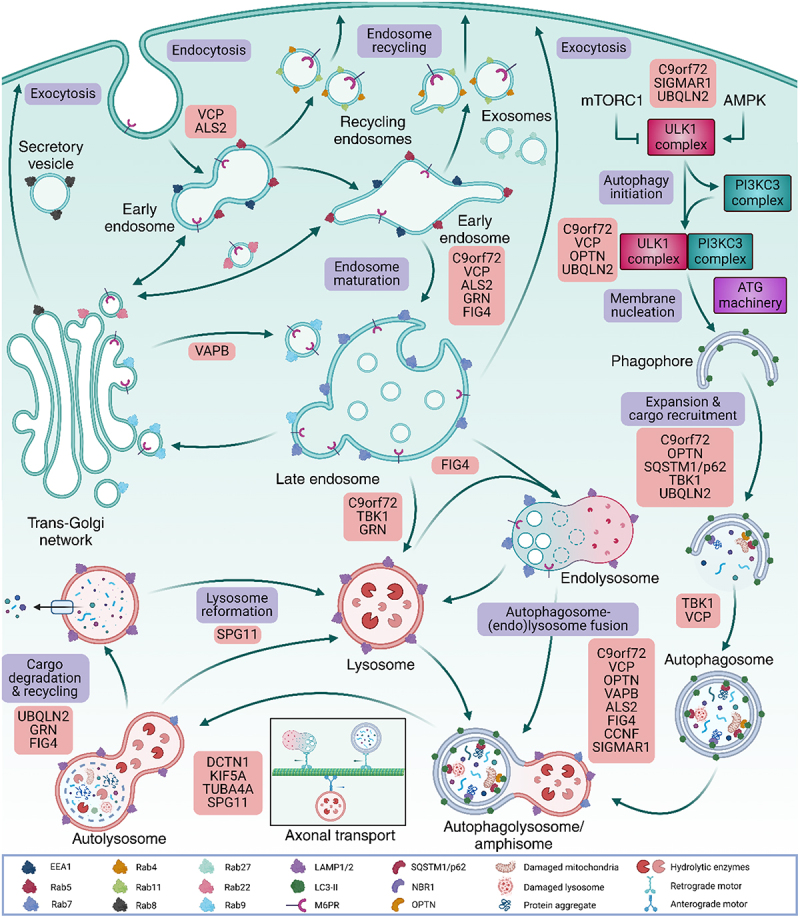
Comprehensive overview of the endolysosomal and autophagy pathways and the specific parts of these pathways that are affected by ALS -or FTD-related genes and risk factors. Various gene variants causative for ALS and/or FTD (depicted in red) have an impact on the endolysosomal and autophagy pathway at different levels (depicted in purple). Abbreviations: AMPK: AMP-activated protein kinase; ATG: autophagy-related; LAMP1/2: lysosomal associated membrane protein 1/2; MAP1LC3-II/LC3-II: microtubule associated protein 1 light chain 3 (lipid modified); M6PR: mannose-6-phosphate receptor; mTORC1: mechanistic target of rapamycin kinase complex 1; NBR1: NBR1 Autophagy Cargo Receptor; OPTN: optineurin; PI3KC3: phosphatidylinositol 3-kinase catalytic subunit type 3; SQSTM1/p62: sequestome 1; TFEB: transcription factor EB; ULK1: unc-51 like autophagy activating kinase 1.

**Figure 3.**
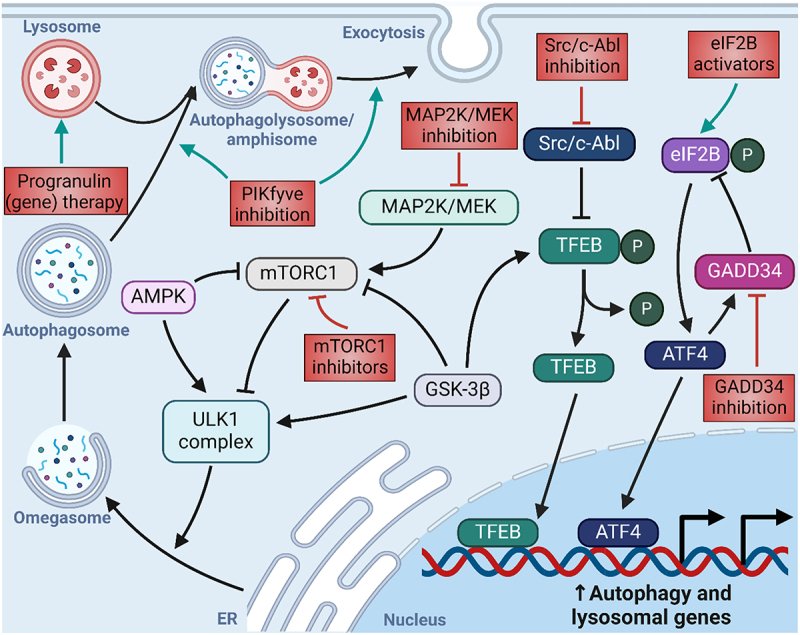
Therapies currently being tested in clinical trials for ALS and/or FTD. Abbreviations: AMPK: AMP-activated protein kinase; ATF4: activating transcription factor 4; eIF2B: eukaryotic translation initiation factor 2B; GADD34: growth arrest and DNA damage-inducible protein; GSK-3β: glycogen synthase kinase-3β; MAP2K/MEK: mitogen-activated protein kinase; mTORC1: mechanistic target of rapamycin kinase complex 1; TFEB: transcription factor EB; ULK1: unc-51 like autophagy activating kinase 1.

While recent overviews of the clinical trial landscape [23] and antisense therapies [487] in ALS have been published recently, we will use the following sections to discuss the promising pipeline of clinical trials and preclinical methods that involve modulation of the autophagy and/or endolysosomal pathways and the inherent challenges that are associated with modulation of the ALP (Figure 2). The overview given in Figure 3 exemplifies the immense complexity of the autophagy and endolysosomal pathways and mutations that impact different stages of those pathways even within the ALS-FTD spectrum. At this stage, it is difficult to envision how to best modulate autophagy as we don’t know enough about how the ALP is altered in different subtypes of ALS and FTD. Therefore, it is not only crucial to further identify the specific stage(s) of the pathways that are defective in each ALS/FTD subtype, but also indispensable to stratify patients by genotype in future clinical trials. For example, enhancing autophagosome formation may have therapeutic potential when patients present with defects early in the autophagy pathway, but no effect when defects in later stages of the pathway are present. In case of defects in later stages of the autophagy pathway (i.e. autophagosome-lysosome fusion, lysosomal clearance, etc.), boosting autophagosome formation may not be suitable and could even aggravate the problems as this would lead to toxicity by the accumulation of autophagosomes that may serve as a platform for the intracellular death-inducing signalling complex (iDISC) that recruits caspase-8 to initiate apoptosis [488]. Since most ALS/FTD-linked mutations impact various stages of the ALP, combinatorial approaches that combine drugs working at different stages of the pathway may be needed to restore autophagic flux as each drug may have an additive beneficial effect. However, the use of combinatorial approaches has the downside of inducing more potential adverse side effects which has already been a major problem in the past when modulating the ALP since most drugs target important signalling nodes (i.e. mTORC1, ULK1, etc.) that also affect other cellular pathways. In addition, timing of ALP treatments should also be considered as some drugs may be beneficial at early disease stages but toxic at later stages and *vice versa*. An example that perfectly illustrates this complexity is the treatment of SOD1^G93A^ mice with trehalose which promotes autophagy in a TFEB-dependent manner [489,490]. While treatment with this natural disaccharide delayed disease onset, initially alleviated motor deficiency and concomitantly reduced mutant SOD1 and p62 levels, trehalose treatment failed to delay further disease progression after reaching a certain threshold and was ultimately not able to improve survival in these mice [490]. In MN, autophagy is essential for NMJ maintenance in the early stages of the disease, but may eventually aggravate disease progression through non-cell-autonomous disease mechanisms in later disease stages [165]. For example, mouse models for *SOD1*-ALS that have an additional TBK1 mutation showed increased autophagy dysfunction and faster muscle denervation at early stages of the disease, but managed to reduce neuroinflammation and improve survival at later stages [134,135]. Overexpression of SQSTM1/p62 in SOD1^H46R^ ALS mice accelerated disease onset, but slowed down disease progression and had little to no impact on neuronal survival, but had a more profound effect on the surrounding glial cells [187]. Although BECN downregulation had a positive effect on survival in the aggressive SOD1^G86R^ transgenic ALS mice, a similar study that investigated the effects of heterozygous BECN1 deletion in two other, less aggressive SOD1 ALS mouse models (SOD1^G127X^ and SOD1^G93A^), conversely reported an exacerbation of the disease upon BECN reduction marked by an increase in disease onset, disease progression, MN degeneration and a significant decrease in survival [491]. This suggests that even within one genetic subtype of ALS, different levels of manipulation of the ALP at the same level can have a differential impact. The efficacy of some treatments may attenuate with disease progression (reviewed in [492]). Therefore, we may need to consider clinical trial designs and genetic testing strategies that allow the administration of therapies to prevent or slow down initial disease manifestations. In recent years, substantial progress has been made in our understanding of autophagic and endolysosomal defects in NDs including ALS and FTD. However, the amount of drugs moving to clinical trials and the translational success rates remain low (Table 2) [493]. This may be in part caused by a lack of understanding the dynamics of the ALP, especially during disease progression and hence the inability to correctly define a specific treatment window for specific ALP targets. Moreover, we are also in dire need of effective methods to measure (cell-type specific) target engagement and track autophagic flux *in vivo*. Therefore, novel imaging techniques in combination with suitable biomarkers of ALP dysfunction are indispensable for the translational success of such treatments.

**Table 2.** Overview of drugs currently in clinical trials for ALS and/or FTD that target the ALP.

Drug	Company	Mechanisms of action	ALS/FTD subtype	Development stage	Trial identifier
Trametinib	GENUV	Mitogen-activated protein kinase (MEK/MAP2K) inhibitor that should activate the autophagy-lysosome pathway	ALS	Phase 1/2a	NCT04326283
Trehalose	Seelos Therapeutics	Dissacharadi with neuroprotective properties that acts by clearing protein aggregates, possibly through activation of autophagy.	ALS	Phase 2/3 (completed)	NCT04297683 + NCT 05136885
Apilimod dimesylate	OrphAI Therapeutics	PIKfyve inhibitor	C9orf72-ALS	Phase 2a	NCT05163886
Monepantel	PharmAust	Repurposed veterinary drug, found to have off-target activity, inhibiting an mTORC1 signalling system and thereby reducing protein accumulation.	ALS	Phase 1	NCT04894240
Rapamycin	Azienda Ospedaliero-Universitaria di Modena	Inhibitor of mTORC1 that aims to enhance autophagy	ALS	Phase 2 (completed)	NCT03359538 + EUDRACT 2016-002399-28
Bosutinib	Pfizer Japan Inc./Kyoto University	Repurposed chronic myelogenous leukaemia (CML) drug functioning as a selective inhibitor of Src/c-Abl tyrosine kinase, promoting autophagy	ALS	Phase 2 (completed)	NCT04744532
DNL-343	Denali Therapeutics	eIF2B activator that inhibits the integrated stress response pathway and influences autophagy progression	ALS	Phase 1b	NCT05006352
ABBV-CLS-7262	Calico/AbbVie	eIF2B activator that inhibits the integrated stress response pathway and influences autophagy progression	ALS	Phase 1	NCT04948645
IFB-088	InFlectis BioScience	Inhibition of GADD34, a protein involved in the integrated stress response (ISR) and an important TFEB target, needed to sustain lysosomal biogenesis and enhance autophagic flux during starvation.	ALS	Phase 2	EudraCT 2021-003875-32
AL001	Alector	monoclonal antibody designed to elevate progranulin levels and increase lysosomal health	C9orf72-FTD	Phase 3 (completed)	NCT04374136 + NCT03987295
*DNL593*	Denali Therapeutics	replacement therapy consisting of progranulin protein fused to an antibody fragment that facilitates the receptor-mediated transcytosis of progranulin into the brain.	GRN-FTD	Phase 1/2	NCT05262023
FRM-0334	FORUM Pharmaceuticals	histone deacetylase (HDAC) inhibitor that aims to increase expression of progranulin	GRN-FTD	Phase 2	NCT02149160
PBFT02	Passage Bio	AAV1 vector gene therapy designed to deliver a copy of the human progranulin gene to the brain.	GRN-FTD	Phase 1b	NCT04747431 + EUDRACT 2020-004499-17
LY3884963	Eli Lilly & Co	AAV9 vector gene therapy designed to deliver a copy of the human progranulin gene to the brain.	GRN-FTD	Phase 1/2	NCT04408625
AVB-101	AviadoBio	AAV9 vector gene therapy designed to deliver a copy of the human progranulin gene under control of a neuron-specific promoter to the brain.	GRN-FTD	Phase ½	NCT06064890

### Small molecule modulators of the ALP

As mentioned earlier, targeting upstream signalling hubs and regulators of the ALP such as mTORC1 may cause adverse and unwanted effects due to the intricate intertwinement with other signalling cascades, mainly pathways involved in regulation of the immune system [494]. However, multiple compounds have been found to activate autophagy and show promising results in preclinical studies, especially with regards to TDP-43 pathology [288,489,495]. These small molecule modulators include mTORC1 inhibitors such as rapamycin, spermidine and berberine but also compounds that modulate lysosomal function including tamoxifen, fluphenazine, methotrimeprazine and promethazine and mTORC1-independent autophagy activators such as trehalose, carbamazepine, lithium carbonate and trametinib that work by altering TFEB and AMPK signalling, inositol signalling or modulation of the GSK-3β (glycogen synthase kinase-3β) or mitogen-activated protein kinase (MAP2K/MEK) pathways respectively [288,489,495-497]. We would like to highlight the use of TFEB modulators which have the potential to simultaneously induce autophagosome synthesis and improve lysosomal function [85,86,498]. As such, several groups have and are currently pursuing this therapeutic avenue and are evaluating the efficacy of (natural) small-molecule TFEB activators in preclinical models of ALS/FTD and other NDs [489,499-502]. Moreover, another intriguing strategy that could increase nuclear TFEB levels and hence its activity is the use of selective inhibitors of nuclear export (SINEs) which was able to activate autophagy and significantly increase lifespan in *C. elegans* [503]. Moreover, recent studies have identified altered signalling of the Src/c-Abl tyrosine kinase in several NDs, including ALS. Overactivation of Src/c-Abl was found to reduce autophagic flux and negatively affect the lysosomal pathway mediated, at least in part, through reduced TFEB signalling [504,505]. Bosutinib, a selective inhibitor of this kinase was found to promote autophagy and restore the autophagic flux in preclinical models [505]. Just recently, an open-label phase 2 clinical trial on bosutinib sponsored by Pfizer Japan (NCT04744532) was successfully completed with acceptable safety and tolerability [506]. Finally, PIKfyve inhibitors show promise in preclinical studies in multiple forms of ALS/FTD is currently being tested in a phase 2a clinical trial [235,507-509], but also here caution is warranted. As mentioned earlier, PIKfyve is a kinase that catalyzes the conversion of PI3P to PI [3,5]P2 which is present on endolysosomal and autophagosomal membranes and thereby acts as a regulator of vesicle fusion [333,335-337,510]. PIKfyve inhibitors such as Apilimod were found to not only enhance autophagosome and lysosome formation and increase early endosome numbers, but also activate an alternative proteostasis mechanism that involves the exocytosis of aggregated proteins in models of sporadic, *FUS-, TDP43*- and *C9orf72*-ALS [235,507,508,510-512]. However, by increasing PI3P levels in the cell, PIKfyve inhibitors prevent Rab5 to Rab7 conversion and hence early endosome maturation [235,507,508]. Moreover, PIKfyve inhibition might negatively affect autophagosome-lysosome fusion, driving cells to pursue other methods such as secretory autophagy to eliminate protein aggregates [511,512]. While this approach protects the cell from the neurotoxic effects of the protein aggregates, it might actually worsen disease progression if these exosomes get taken up by healthy cells. In addition, prolonged treatment with PIKfyve inhibitors could lead to the accumulation of enlarged early endosomes, a trigger known to induce TDP-43 proteinopathy and cause neurodegeneration [106,335]. Therefore, it is unclear whether the initial neuroprotective effect of PIKfyve inhibition is persistent or rather short-lived as it could eventually be counteracted by its detrimental effects on neurotoxic protein propagation and endosomal maturation [67,106,511].

Another emerging therapeutic approach are the so called “synthetic molecular glues and protein degraders” [513,514]. These molecular glues are small molecules that stabilise (specific) protein-protein interactions and could therefore by used to specifically increase the affinity of neurotoxic proteins with components of the proteostasis network such as LC3, p62, or components of the UPS. Protein degraders work by targeting specific proteins for degradation by the proteasome or autophagolysosome. Focusing on the latter pathway, this can be achieved by either therapeutic antibodies, AuTophagosome-TEthering Compounds (ATTECs) or AUtophagy TArgeting Chimeras (AUTACs). While ATTECs work by directly tethering specific target protein to the autophagosome membrane which are then delivered to the lysosome for degradation, AUTACs indirectly mark specific target proteins with a tag that serves as a degradation signal, mimicking endogenous autophagy signalling pathways [513,514].

With respect to FTD, the translation of ALP-targeting small molecules into the clinic remains rather sparse. Similar to the genetic therapies tested for FTD (see below), several therapeutic strategies for FTD try to restore or increase PGRN levels and hence ameliorate lysosomal health (Table 2). Clinical trials sponsored by Alector, Denali Therapeutics and FORUM Pharmaceuticals aim to achieve this by using a monoclonal antibody designed to elevate progranulin levels, a replacement therapy consisting of progranulin protein fused to an antibody fragment that facilitates the receptor-mediated transcytosis of progranulin into the brain or the use of an histone deacetylase (HDAC) inhibitor in order to increase expression of progranulin, respectively.

### Genetic therapies to target ALP dysfunction in ALS/FTD

Genetic therapies are further classified into gene silencing therapies that aim to inhibit the expression of toxic genes by targeting their mRNAs with RNA interference (RNAi) or antisense oligonucleotides (ASOs), gene editing therapies that utilise the CRISPR–Cas9 gene editing machinery or other programmable nucleases to either correct or introduce pathogenic gene mutations and lastly gene replacement therapies that comprise the use of (viral) vectors to deliver functional copies of mutant genes [515-517]. ASOs are chemically modified single-stranded oligonucleotides designed to specifically target certain mRNAs. By doing so, they can either attract RNAse H and degrade the target mRNA which holds promise for conditions where a toxic gain-of-function is primarily involved. Depending on the modifications of the ASO, binding to its target can alter processing of the mRNA and hence modulate translation, localisation or alternative splicing of its target, a strategy suitable to tackle some loss-of-function mechanisms (reviewed in Van Daele et al. 2023) [487]. We previously mentioned the unprecedented success of QALSODY® (Tofersen) for the treatment of *SOD1*-ALS patients and a FUS-targeting ALSO seems to hold promise as well [487,518,519]. The first attempts to target sense repeat containing transcripts in ALS-FTD patients with a *C9orf72* repeat expansion were not successful, although the production of several DPRs was clearly reduced [520]. Nevertheless, ASOs are also being designed to target specific molecular pathways affected in ALS/FTD. One such example is PIKfyve ASO-mediated silencing by AS-202. Similar to the results obtained with small-molecule inhibitors of the kinase, PIKfyve silencing rescues neurodegeneration in iPSC-derived MNs [511]. CRISPR/Cas9-based gene editing are the most recent addition to the pool of gene therapies and although they have opened up a whole new window of therapeutic opportunities, substantial progress in terms of safety and efficacy is still needed to optimise this kind of therapy for clinical use. Nevertheless, preclinical studies on *C9orf72* ALS/FTD patient iPSC-MNs underscored the therapeutic potential of CRISPR-mediated gene alterations as a recent study could successfully delete part of the *C9orf72* promotor region and hence reduce the production of toxic DPR proteins and rescue neurodegeneration [521]. Alternatively, a promising study explored the use of the RNA-targeting CRISPR-Cas13 system for the treatment of *C9orf72* ALS/FTD [522]. Using this approach, multiple RNA transcripts can be successfully targeted using an adeno-associated viral (AAV) vector. While this therapy holds tremendous promise for its use in *C9orf72* ALS/FTD and possibly other diseases characterised by toxic RNA gain-of-function mechanisms, further research on its long-term efficiency, off-target effects and safety are needed. Finally, gene replacement therapies hold great potential for ALS/FTD subtypes mainly caused by pathogenic loss-of-function mechanisms. While several ALS-associated genes could potentially benefit from such treatment (reviewed in detail by Giovannelli et al., 2023), there are no ongoing clinical trials for ALS [515]. In contrast, three separate and promising clinical trials for GRN-FTD are underway to explore the use of AAV-mediated delivery of human progranulin genes to the brain in order to restore progranulin expression. Interim results from one of those studies revealed that treatment with the investigational gene therapy showed good efficacy in preclinical cellular and rodent models of *GRN*-FTD and was found to be generally safe and relatively well-tolerated in non-human primates and patients suffering from *GRN*-FTD [523]. However, whether it can also boost lysosomal function and the outcome of patients requires further study.

## Concluding remarks and future prospectives

Decades of research have established a profound link between alterations of the autophagy and endolysosomal pathways and ALS/FTD pathogenesis. The known role of autophagy in aggregate removal has been known for a long time, but the insights into the role of endolysosomal dysfunctions are emerging more recently and have often been overlooked in the past. Several recent studies underscoring the inherent relationship between endolysosomal system dysfunctions and the development of TDP-43 proteinopathy, highlight the importance of this pathway, which will hopefully provide novel therapeutic targets in the upcoming years. A better and more profound understanding of each part of the autophagic pathway (in neurons, but also in different supporting glial cells) in the different genetic subtypes of ALS/FTD is required for the development of effective therapies. The advent of molecular strategies to combat ALS and FTD has really been a beacon of hope for many patients suffering from these devastating diseases. New therapeutic approaches intervening with autophagy and the endolysosomal pathways will ultimately bring clarity about the importance of these pathways for ALS/FTD.

## Abbreviations

AD: Alzheimer’s Disease; ALS: Amyotrophic Lateral Sclerosis; ALS2: Alsin; ALP: Autophagy-Lysosome Pathway; AMPK: AMP-Activated Protein Kinase; ASO: Antisense Oligonucleotide; ATG: Autophagy-Related; ATTEC: AuTophagosome-TEthering Compound; AUTAC: AUtophagy TArgeting Chimera; AAV: Adeno-Associated Virus; C9orf72: Chromosome 9 Open Reading Frame 72; CCNF: Cyclin F; CHMP2B: Charged Multivesicular Body Protein 2B; CMT: Charcot-Marie-Tooth Disease; DCTN1: Dynactin 1; DPR: Dipeptide Repeat Protein; EEA1: Early Endosome Antigen 1; ERAD: Endoplasmic Reticulum-Associated Degradation; ER-phagy: Endoplasmic Reticulum Autophagy; ESCRT: Endosomal Sorting Complex Required for Transport; FIP200: Focal Adhesion Kinase Family Interacting Protein of 200 kDa; FTD: Frontotemporal Dementia; FUS: FUS DNA Binding Protein; GAP: GTPase-Activating Protein; GEF: Guanine Exchange Factor; GRN: Granulin; GSK-3β: Glycogen Synthase Kinase 3 Beta; HD: Huntington’s Disease; HDAC: Histone Deacetylase; HOPS: Homotypic Fusion and Protein Sorting; HRE: Hexanucleotide Repeat Expansion; HSP: Hereditary Spastic Paraplegia; HSP70: Heat Shock Protein 70; IBMPDD: Inclusion Body Myopathy Associated with Paget Disease of Bone and Frontotemporal Dementia; iDISC: Intracellular Death-Inducing Signaling Complex; iPSC: Induced Pluripotent Stem Cells; KIF5A: Kinesin Heavy Chain Isoform 5A; LAMP1: Lysosomal-Associated Membrane Protein 1; LC3: Microtubule-Associated Protein 1 Light Chain 3; LIR: LC3-Interacting Region; MAP2K/MEK: Mitogen-Activated Protein Kinase Kinase; MN: Motor Neuron; MPR: Mannose-6-Phosphate Receptor; MVB: Multivesicular Body; mTORC1: Mechanistic Target of Rapamycin Kinase Complex 1; NBR1: Neighbor of BRCA1; NCL: Neuronal Ceroid Lipofuscinosis; ND: Neurodegenerative Disease; NDP52: Nuclear Domain 10 Protein 52; OPTN: Optineurin; PDB: Paget’s Disease of Bone; PGRN: Progranulin; PI(3,5)P2: Phosphatidylinositol 3,5-Bisphosphate; PI(3)P: Phosphatidylinositol 3-Phosphate; PI4P: Phosphatidylinositol 4-Phosphate; PtdIns3K: Class III Phosphatidylinositol 3-Kinase; PTEN: Phosphatase and Tensin Homolog; RAN: Repeat-Associated Non-AUG; RNAi: RNA Interference; SINEs: Selective Inhibitors of Nuclear Export; SigR1: Sigma Receptor 1; SMCR8: Smith-Magenis Syndrome Chromosome Region Candidate Gene 8; SNARE: Soluble N-Ethylmaleimide-Sensitive Factor Attachment Protein Receptor; SOD1: Superoxide Dismutase 1; SPG11: Spastic Paraplegia 11; SQSTM1/p62: Sequestosome 1/p62; TANK: TANK Binding Kinase 1; TARDBP/TDP-43: TAR DNA Binding Protein 43; TBK1: TANK Binding Kinase 1; TFEB: Transcription Factor EB; TMEM106B: Transmembrane Protein 106B; TUBA4A: Tubulin α4A Chain; ULK1: Unc-51 Like Autophagy Activating Kinase 1; UPS: Ubiquitin-Proteasome System; VAPB: Vesicle-Associated Membrane Protein-Associated Protein B; VCP/p97: Valosin Containing Protein.
